# Fatigue and Fracture Resistance Testing of Polyether Ether Ketone (PEEK) Implant Abutments in an Ex Vivo Chewing Simulator Model

**DOI:** 10.3390/ma15196927

**Published:** 2022-10-06

**Authors:** Babak Saravi, Anselm Flohr, Sebastian B. Patzelt, Benedikt C. Spies, Derek Hazard, Ralf J. Kohal

**Affiliations:** 1 Department of Orthopedics and Trauma Surgery, Faculty of Medicine, University of Freiburg, Medical Center—University of Freiburg, Hugstetter Street 55, 79106 Freiburg, Germany; 2 Department of Prosthetic Dentistry, Faculty of Medicine, Medical Center—University of Freiburg, Center for Dental Medicine, University of Freiburg, Hugstetter Street 55, 79106 Freiburg, Germany; 3 Private Dental Clinic, Am Dorfplatz 3, 78658 Zimmern ob Rottweil, Germany; 4 Institute of Medical Biometry and Medical Statistics, Faculty of Medicine, Medical Center—University of Freiburg, University of Freiburg, Hugstetter Street 55, 79106 Freiburg, Germany

**Keywords:** PEEK, abutments, zirconia, dental implant, fracture resistance, loading, ex vivo, prosthetics

## Abstract

Polyether ether ketone (PEEK) has been introduced into implant dentistry as a viable alternative to current implant abutment materials. However, data on its physico-mechanical properties are still scarce. The present study sought to shed light on this topic utilizing an ex vivo chewing simulator model. A total of 48 titanium two-piece implants were allocated into three groups (*n =* 16 per group): (1) implants with PEEK abutments and an internal butt-joint connection (PBJ), (2) implants with PEEK abutments and an internal conical implant–abutment connection (PC), and (3) implants with zirconia abutments and an internal butt-joint connection (ZA). All abutments were restored with a non-precious metal alloy crown mimicking the upper right central incisor. A dynamic chewing simulation of half (*n* = 8) of the specimens per group was performed with 5 × 106 cycles and a load of 49 N at a frequency of 1.7 Hz with thermocycling between 5 and 55 °C. The other eight specimens served as unloaded controls. Surface roughness, implant–abutment connection microgaps (IACMs), and the titanium base–abutment interface microgaps (TAIMs) in the loaded groups were evaluated. Finally, a quasi-static loading test was performed in a universal testing machine with all samples to evaluate fracture resistance. Overall, 23 samples survived the artificial chewing process. One abutment screw fracture was observed in the PC group. The ZA group showed higher surface roughness values than PEEK abutments. Furthermore, ZA revealed lower TAIM values compared to PEEK abutments. Similarly, ZA was associated with lower IACM values compared to PBJ. Fracture loads/bending moments were 1018 N/704 N cm for PBJ, 966 N/676 N cm for PC, and 738 N/508 N cm for ZA, with no significant differences compared to the unloaded references. Artificial loading did not significantly affect fracture resistance of the examined materials. PEEK abutments were associated with better load-bearing properties than zirconia abutments, although they showed higher microgap values. PEEK abutments could, therefore, be feasible alternatives to zirconia abutments based on the present ex vivo findings resembling 20 years of clinical service.

## 1. Introduction

Implant-based therapies have become a well-established treatment approach in modern dentistry. Osseointegration of oral implants is generally considered to be the main key to long-term clinical success. However, the functional and structural connection between implant and bone is not the only critical factor in clinics. Superstructures of oral implants, including the complex of abutments, crowns, and their interfaces, as well as their bonds with bone-anchored implants, affect the mechanical behavior and, therefore, the clinical success [1,2,3]. The influence of material and geometry on physical properties is not only of interest from a biomechanical point of view. Seeking advanced materials and improved geometries could also lead to a better therapeutical option for affected patients in the future if sufficient evidence is provided. An advantageous way to fulfill this task is by testing promising and novel materials in an appropriate environment simulating in vivo conditions as close as possible. Accordingly, our workgroup implemented an ex vivo chewing simulator model, which was able to account for the complex mechanical and thermal conditions existent in the oral cavity [4,5]. Moreover, it allowed comparing different experimental groups simultaneously, reducing potential bias.

The absence of periodontal ligaments reduces tactile sensitivity compared to a healthy periodontium, which can lead to increased force peaks on the restoration and, thus, to fractures of the abutment material [6]. Consequently, superstructure materials should have satisfying mechanical properties to withstand the chewing forces. In addition, implant abutments require improved esthetic features when applied in esthetic areas [7]. With regard to the aforementioned requirements, the use of abutments made of zirconia has become widespread in recent years, backed by evidence from several clinical studies [8,9]. Survival, technical, and biological complication rates of zirconia abutments are comparable to titanium abutments for up to 5 years of exposure in the posterior region, although titanium abutments were reported to have a better mechanical resistance in a recent systematic review [8,10]. However, the zirconia material also has some disadvantages such as brittleness and aging in an aqueous environment [11]. These can reduce stability and increase the risk of material failure [12]. Therefore, materials having mechanical properties of metals, aesthetic features of ceramics, and tooth-like elasticity are still highly warranted.

Polyether ether ketone (PEEK) has been introduced as a high-performance thermoplastic polymer implant material in the 1990s [13,14]. PEEK has many advantages, including good mechanical and esthetic properties and good biocompatibility, as it is considered biologically inert [15,16]. Its composite structure can be altered to adjust flexural strength and modulus of elasticity for dental applications [17]. Although its biological inertness might be the greatest challenge for its application as an oral implant material [18], its application as an implant abutment is reasonable [19,20]. Currently, however, PEEK has so far only been used as a temporary abutment material [21,22,23]. In an in vitro study by Rosentritt et al. [24], eight PEEK abutments on titanium adhesive bases were restored with zirconia anterior crowns. They were then subjected to artificial masticatory loading of 3.6 million masticatory cycles and 10 N under thermocycling between 5 and 55 °C with zirconia abutments as the control group. No test specimen survived the artificial chewing simulation for more than 1 million cycles. However, the reason for this was not a direct failure of the PEEK abutments but was caused by screw loosening and screw fracture during loading. A similarly designed study used PEEK as a framework material for single molar crowns manually veneered with a composite material [25]. Subsequently, the test specimens were loaded with 50 N for 1.2 million cycles under thermocycling between 5 and 55 °C. Implant-supported restorations were investigated in two groups, with the crowns of one group placed directly on the implant screw-retained abutments. In the other group, the crowns were provided with a transocclusal canal, bonded to the abutments in the laboratory, and only then screwed to the implant via the canal. This channel was then sealed with composite. No statement was made about the implant–abutment connection type and the abutment material. It could be assumed that the abutments were hybrid abutments with titanium adhesive bases. In the third group, veneered PEEK crowns were cemented to natural teeth. All test specimens survived the cyclic chewing simulation in the two cemented groups. Subsequently, these two groups were subjected to a static load test in a universal testing machine. The mean fracture force values were 921 N (abutment group) and 747 N (tooth group). In the transocclusal screw-retained abutment group, cracks occurred in the veneer of all test specimens during the chewing simulation, which had to be considered a failure [25]. Failure of the PEEK frameworks was not reported. The aforementioned studies showed that PEEK remained stable after the simulated dynamic chewing simulation. However, the tests only covered a simulation of approximately 2–5 years. In addition, the studies only investigated fracture strength. Other changes, such as micro- and macroscopic surface changes that may have occurred during the chewing simulation, were not investigated in the PEEK abutments. However, it seems reasonable to check whether the surface remains unchanged in integrity after continuous mechanical loading before long-term clinical use [26].

Considering the promising features of PEEK materials and the lack of available evidence in this field, we sought to evaluate the mechanical properties of PEEK abutments with two different internal geometries on two-piece titanium oral implants and compare them to an established zirconia abutment. To maintain a comparable clinical setting, we tested the aforementioned materials in an ex vivo chewing simulator model.

## 2. Materials and Methods

### 2.1. Experimental Setup

**Figure 1:**
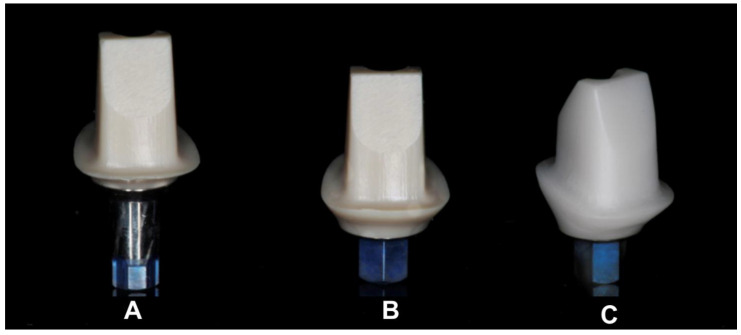
PC abutment (**A**); PBJ abutment (**B**); ZA abutment (**C**).

**Figure 2:**
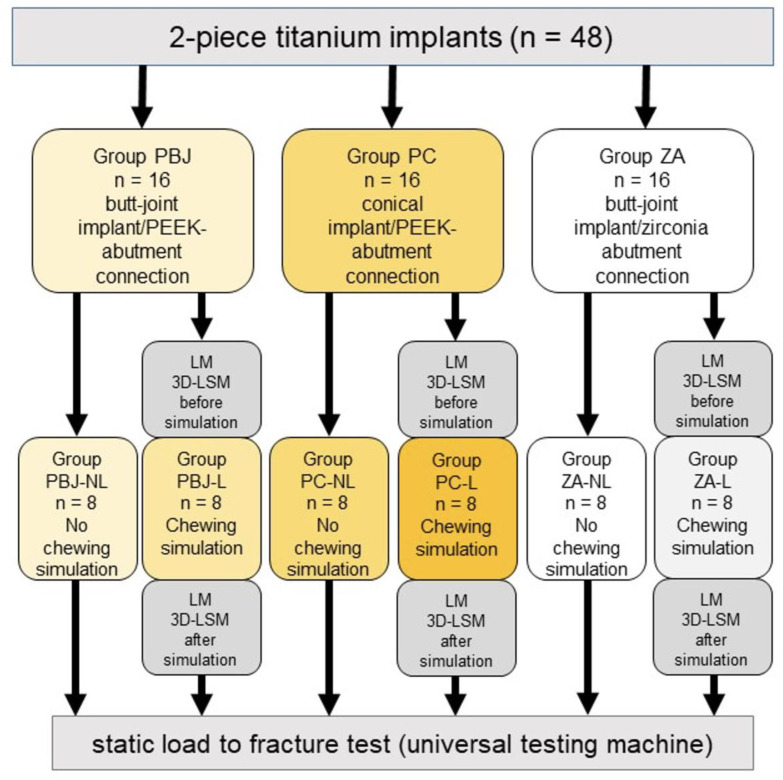
Flowchart of the study groups. Experimental groups (-L) underwent pre- and post-loading examinations for comparative analysis. LM: light microscopy; 3D-LSM: 3D laser scanning microscopy; PBJ: PEEK abutments with an internal butt-joint connection; PC: PEEK abutments with an internal conical implant–abutment connection; ZA: zirconia abutments.

The present study examined a total of 48 two-piece titanium-based oral implants with screw-retained abutments restored with cobalt–chromium alloy crowns. The experimental setup consisted of three groups to compare the effect of the internal abutment geometry and abutment material on physico-mechanical outcomes: (1) 16 PEEK abutments (P2F, SIC invent AG, Basel, Switzerland) on a titanium base with an internal hexagonal butt-joint connection to the implant (PBJ) (SICmax, SIC invent AG, Basel, Switzerland) (Figure 1A), (2) 16 PEEK abutments (P2F, SIC invent AG) on a titanium base (SIC invent AG, Basel, Switzerland) with an internal conical connection to the implant (PC) (SICvantage max, SIC invent AG, Basel, Switzerland) (Figure 1B), and Y-TZP zirconia abutments on a titanium base with internal hexagonal butt-joint connection (ZA) (SICmax, SIC invent AG, Basel, Switzerland) (Figure 1C). The implants in the PBJ group (SICmax; SIC invent AG, Basel, Switzerland) were grade 4 pure titanium implants with a length of 13 mm and a diameter of 3.7 mm. The SICmax implants utilized in the PBJ group were also used in the ZA group. The implants in the PC group were also pure titanium grade 4 implants (SICvantage; SIC invent AG, Basel, Switzerland). These implants had the same outer geometry and dimensions (length = 13 mm, diameter = 3.7 mm) as SICmax implants.

Eight specimens of each group underwent a dynamical loading (L) procedure and were subsequently submitted to a static fracture load (PBJ-L, PC-L, ZA-L). The remaining eight specimens in each group served as controls and were not artificially loaded (NL) but directly loaded to fracture in a universal testing machine (PBJ-NL, PC-NL, ZA-NL). The utilized titanium implants had the same length (13 mm) and diameter (3.7 mm). ZA abutments were bonded to titanium bases (SIC invent AG) using the resin cement Panavia V5 (Kuraray Noritake Dental Inc., Tokyo, Japan) before the start of the experiments. In contrast, PBJ and PC were already pressed on titanium bases by the supplier and were not further processed. PBJ and ZA had an internal hexagon with a long-walled guide surface, whereas PC had a self-locking morse taper inner connection with a cone angle of 2.8 degrees. Abutments were screw-retained on their respective implants using a torque of 20 Ncm. Subsequently, all samples were provided with cobalt–chromium crowns resembling an upper right central incisor (Simeda, Frankfurt, Germany) using standardized cementation protocols for PEEK and zirconia abutments, according to the manufacturers. For the attachment of the cobalt–chromium crowns to the zirconia abutments, the contact surfaces of the zirconia abutments and the inner surfaces of the cobalt–chromium crowns were blasted with corundum (1.5 bar, 50 µm Picodent precious corundum). Ceramic Primer Plus was thinly applied to the zirconia abutment and the crown, and Panavia V5 (Kuraray Noritake Dental Inc., Tokyo, Japan) was used to bond them together. The abutments and the crowns were also sandblasted for the cementation of the cobalt–chromium crowns onto the PEEK abutments. The abutments and the crowns were subsequently primed with Visio.link (abutment) and MKZ-Primer (crown) (both Bredent, Senden, Germany). The adhesive cementation was finally performed with DTK-Adhesive (Bredent). Cementing aids were used to adhere the crowns to the abutments during the curing process. The experimental setup is illustrated in Figure 2.

All samples were embedded according to ISO 14801 in standardized tubes made from PEEK (length: 20 mm; diameter: 16 mm) using LuxaCore Automix Dual (DMG, Hamburg, Germany) to mimic osseointegration. To simulate a bone loss of 3 mm, implant shoulders were placed 3 mm above the surface of the tubes. Subsequently, eight samples of all three groups to be dynamically loaded (PBJ-L, PC-L, and ZA-L) were then transferred to aluminum sample holders with an angle of 30° to the vertical axis to simulate a physiological bite situation. The samples then underwent a dynamic loading procedure in a dual-axis chewing simulator (CS-4.8, SD Mechatronik, Feldkirchen-Westerham, Germany) with 5 million chewing cycles at a load of 49 N and a chewing frequency of 1.7 Hz. A thermocycling between 5 °C and 55 °C was also initiated. Assuming a masticatory rate of 250,000 contacts/year [27], this artificial setup correlated with a clinical exposure of approximately 20 years. The settings, water temperature, tooth contacts, and status of the specimens were checked twice per day, and any occurrences were recorded.

### 2.2. Outcome Measurements

Implant–abutment connection microgaps (IACMs) and titanium base–abutment interface microgaps (TAIMs) (in µm) in all experimental groups were investigated before and after dynamic loading using light microscopy (Olympus SZH 10, Olympus, Tokyo, Japan). A standardized protocol was established for measurements: a total of 72 circumferential measurements were performed per specimen for IACMs and TAIMs (*n* = 12 positions; *n* = 6 measurements per position). The samples were fixed in an individualized apparatus, allowing a 360° rotation with a 12-position click wheel to standardize the measurements. Pictures were made with 70-fold magnification, and the gaps were measured using the Cell^P software (Olympus, Tokyo, Japan).

All experimental group samples undergoing chewing simulation underwent an in-depth surface analysis using 3D laser scanning microscopy (VK X 200 3D-LSM, Keyence, Tokyo, Japan). Measurements were performed at defined areas before and after dynamic loading using a 1000-fold magnification and a standardized protocol for positioning specimens. The average surface roughness “Ra” (µm) and the average surface profile maximal heights “Rz” (µm) were determined as outcomes [28]. The four main areas of measurement were at the “3, 6, 9, and 12 o‘clock” positions of the specimens, with the 6 o’clock position located at the palatal aspect of the abutment (loading side) and the 12 o’clock position at the labial aspect of the abutment.

All dynamically loaded samples that survived the chewing simulation process (PBJ-L, PC-L, and ZA-L) and all not-dynamically loaded control samples (PBJ-NL, PC-NL, and ZA-NL) were subjected to a final static loading procedure utilizing a universal testing machine (Z010/TN2S, ZwickRoell, Ulm, Germany) to determine fracture resistance of the implant/abutment/crown complexes. Equivalent to the dynamic loading in the chewing simulator, all samples were fixed in aluminum sample holders at an angle of 30 degrees to the vertical. A force was applied at a crosshead speed of 2 mm/min until a fracture of the sample occurred. An XY-writer recorded the force–deformation diagram (Spare 2000 Kipp&Zonen, Delft, The Netherlands). The bending moments at fracture were documented for group comparisons.

### 2.3. Statistics

For descriptive statistics, the medians, means, and standard deviations were calculated for the parameters IACM and TAIM, surface roughness Ra and Rz, and bending moment. These parameters were then analyzed utilizing linear regression models, considering the following influencing variables: material (PEEK/ZrO2), implant–abutment geometry (butt-joint vs. conical), and chewing simulation (=timepoint: before vs. after dynamical loading). Furthermore, an analysis of variance followed by Scheffé’s method for multiple pairwise comparisons was applied. Outcomes were calculated with their 95% confidence intervals (95% CIs). Means and standard deviations are shown in the text, whereas medians are illustrated in box plots with their interquartile ranges (IQRs). A *p*-value < 0.05 was considered statistically significant. The analyses were exploratory in nature. As a result, *p*-values and 95% confidence intervals were not corrected for multiple comparisons and inferences drawn from them were possibly not reproducible. For the power analysis, we initially selected the sample size based on previous similar chewing simulation studies [5,29,30,31,32,33,34,35,36,37,38,39,40,41,42,43], which indicated a range of seven to fifteen specimens in subgroups, with eight specimens being a common sample size [5,42,43]. Using GPower [44], we also conducted a post hoc power analysis to ensure that our study design had sufficient power to detect the effects of the chewing simulation and materials on fracture loads. Notably, pairwise analyses were conducted with 12 measurement positions/group and *n* = 6 measurements/position, resulting in a total of *n* = 72 per group for pairwise statistics. The post hoc power calculation determined that the 72 samples per group would provide a 95% probability of finding a significant difference between the corresponding groups at a 5% level of significance. We conducted this post hoc power analysis while assuming that there was a small effect size of 0.135, as proposed by Cohen (small effect size: 0.1–0.3) [45]. The analysis was performed using Stata Statistical Software Release 14.2 (StataCorp. 2011, College Station, TX, USA).

## 3. Results

### 3.1. Dynamic Loading in the Chewing Simulator

**Figure 3:**
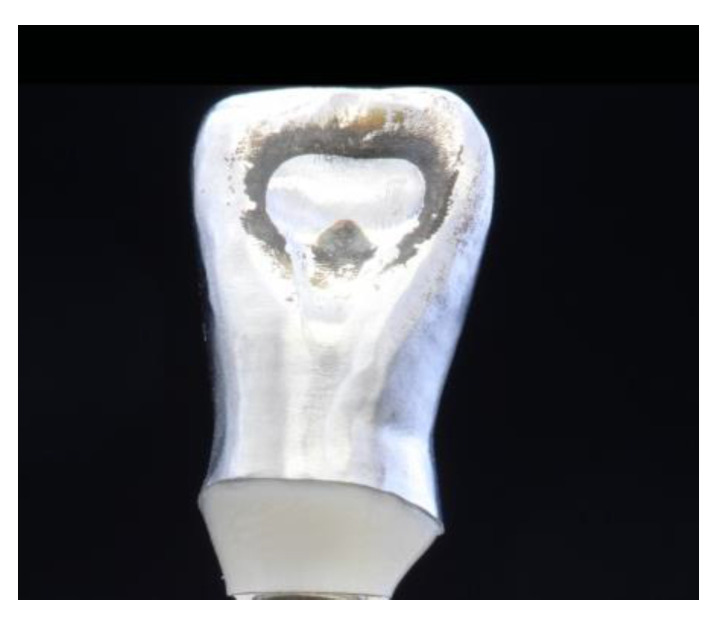
Sample with extensive wear at the contact point of the antagonist.

The specimens were evaluated twice per day during the whole dynamic loading process (5 million cycles), corresponding to 20 years of clinical simulation. After dynamic loading, an extensive wear at the palatal crown aspect (contact point of the antagonist) of all sample crowns was observed (Figure 3). One sample in the PC group showed an abutment screw fracture after approximately 4.6 million dynamic loading cycles. This specimen was regarded as a failure and was considered for statistical analysis with a fracture value of 49 N (load of the chewing simulator). The remaining samples survived the artificial loading process without revealing signs of failure, resulting in a survival rate of 100%, 87.5%, and 100% for PBJ, PC, and ZA, respectively.

### 3.2. Assessment of Implant-Abutment Connection Microgaps (IACMs) and Titanium Base-Abutment Interface Microgaps (TAIMs)

**Figure 4:**
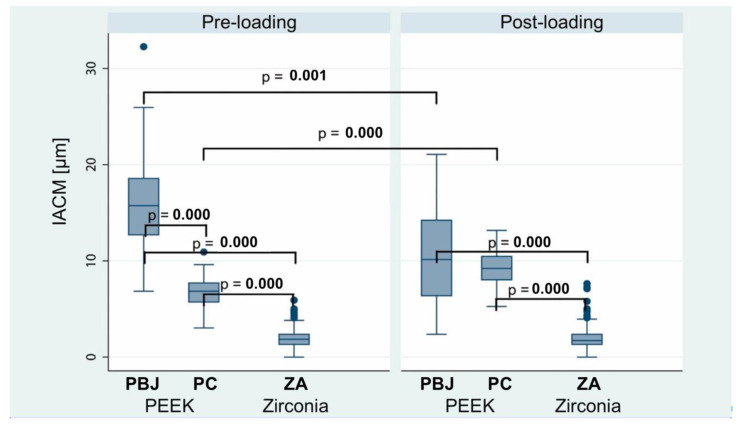
Comparison of implant–abutment connection microgaps (IACMs) (in µm) before and after dynamical loading. Box plots illustrate medians and interquartile ranges of 12 measurement positions/group and *n* = 6 measurements/position, resulting in a total of *n* = 72 per group for statistics. PBJ: PEEK abutments with an internal butt-joint connection; PC: PEEK abutments with an internal conical implant–abutment connection; ZA: zirconia abutments. Only significant differences are indicated by *p*-values.

IACMs and TAIMs were assessed before and after dynamic loading for groups PBJ-L, PC-L, and ZA-L. In both pre- and post-loading evaluations, ZA was associated with significantly lower IACM values than its PEEK comparators (*p* < 0.0001) (Figure 4 and Table 1). Additionally, PC revealed lower IACM values than PBJ prior to dynamical loading (6.8 µm ± 1.5 µm vs. 15.9 µm ± 4.5 µm; *p* < 0.0001). However, this finding was absent after dynamical loading (9.3 µm ± 1.7 µm vs. 10.6 µm ± 5.1 µm; *p* = 0.405). Changes in IACMs caused through the loading procedure for both PEEK groups could be observed, but not for the ZA group. The influence of abutment geometry (PC vs. PBJ) was further analyzed in a linear regression model adjusted for measurement position and timepoint. Results showed that PC were associated with 5.4 µm (95% CI: 4.1–7.6 µm; *p* < 0.0001) lower IACM values than PBJ. Moreover, focus on loading as the dependent variable in the regression model revealed that IACM values increased by 2.5 µm (95% CI: 1.4–3.6 µm; *p* < 0.0001) for PC, whereas PBJ was associated with a decrease of 5.4 µm (95% CI: 2.1–8.6 µm; *p* < 0.0001). The linear regression model showed no significant influence of loading on ZA IACM values (*p* = 0.826).

**Figure 5:**
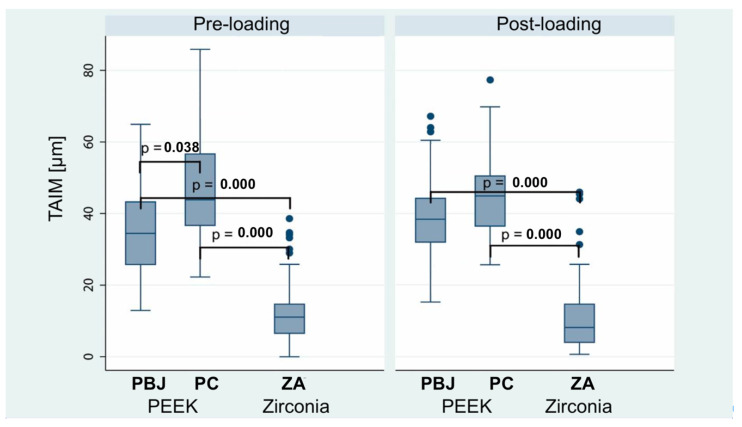
Comparison of titanium base–abutment interface microgaps (TAIMs) (in µm) before and after dynamical loading. Box plots illustrate medians and interquartile ranges of 12 measurement positions/group and *n* = 6 measurements/position, resulting in a total of *n* = 72 per group for statistics. PBJ: PEEK abutments with an internal butt-joint connection; PC: PEEK abutments with an internal conical implant–abutment connection; ZA: zirconia abutments. Only significant differences are indicated by *p*-values.

Similar to the IACM results, ZA was also associated with lower pre- and post-loading TAIM values compared to the PEEK groups (*p* < 0.0001) (Figure 5 and Table 2). Furthermore, PC showed higher TAIM values than PBJ, which was in contrast to IACM findings. However, this finding was not significant after loading (*p* = 0.101). None of the groups showed significant changes in TAIM values caused by the loading procedure. Finally, it was evaluated whether there was a difference in TAIM values comparing pooled PEEK abutments versus zirconia abutments while adjusting for measurement time (pre- and post-loading). As a result, ZA was generally associated with 30.3 µm (95% CI: 25.0–35.6 µm; *p* < 0.0001) lower TAIM values compared to PEEK abutments.

### 3.3. Evaluation of Surface Characteristics

An assessment of the PEEK abutments was performed before and after dynamical loading utilizing light microscopy. A total of 1152 “before-and-after” images were compared. Possible surface changes such as material chippings, changes in topography, and other differences before and after loading were assessed. The PEEK surfaces showed a distinct polygonal surface characteristic that originated from the production process. No surface changes in the light microscopy evaluation could be observed in the post-loading images compared to the pre-loading situation for 47 test specimens. Only the PC sample, which had failed during dynamical loading, showed a defect in the basal–palatal abutment area. Notably, foreign bodies in the TAIM of PEEK samples (*n* = 6/16; 37.5%) were frequently observed. In contrast, they were not seen in the ZA group. 

**Figure 6:**
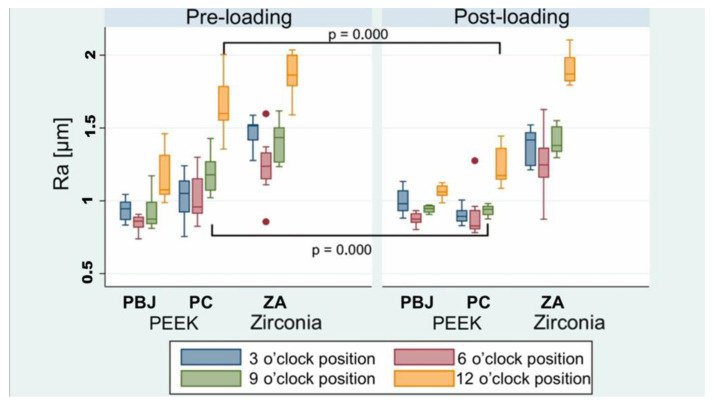
Comparison of pre- and post-loading average surface roughness Ra (in µm) stratified by measurement (clockwise) position. PBJ: PEEK abutments with an internal butt-joint connection; PC: PEEK abutments with an internal conical implant–abutment connection; ZA: zirconia abutments.

Furthermore, an in-depth analysis of surface characteristics was performed using 3D-LSM. Pre- and post-loading Ra values are shown in Table 3. Additionally, Ra value changes were stratified by measurement position (four measurements at the 3, 6, 9, and 12 o’clock positions) (Figure 6). A significant decrease in post-loading Ra values for the 9 o’clock and 12 o’clock measurement positions of PC (*p* < 0.0001) compared to the pre-loading situation was observed. All other comparisons did not reveal significant changes. In addition, zirconia was associated with higher Ra values of approximately 0.5 µm (95% CI: 0.472–9.571 µm; *p* < 0.0001) compared to pooled PEEK abutments.

**Figure 7:**
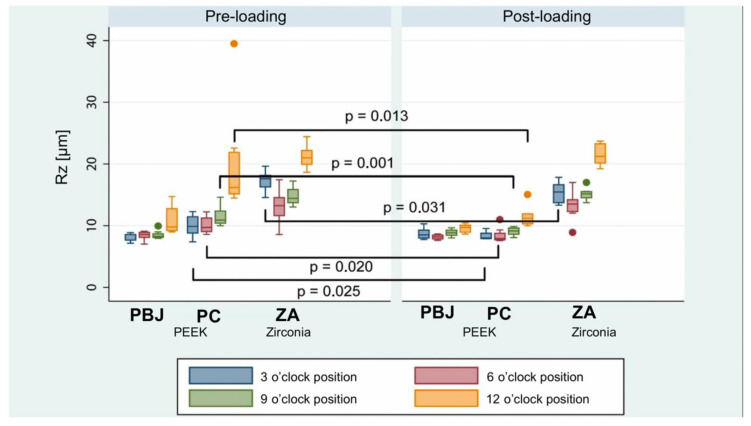
Comparison of pre- and post-loading average surface profile maximal heights Rz (in µm) stratified by measurement position. PBJ: PEEK abutments with an internal butt-joint connection; PC: PEEK abutments with an internal conical implant–abutment connection; ZA: zirconia abutments.

Pre- and post-loading Rz values for all groups are listed in Table 4. Rz value changes were mainly seen in the PC group (Figure 7). There was a significant decrease in Rz in all post-loading PC position measurements compared to the pre-loading situation (*p* < 0.05). Furthermore, the post-loading 3 o’clock ZA showed a lower Rz value compared to the pre-loading situation (*p* < 0.031). In addition, zirconia abutments generally had a 7.6 µm (95% CI: 6.916–8.211µm; *p* < 0.0001) higher Rz value than pooled PEEK abutments. 

### 3.4. Static Loading Test

**Figure 8:**
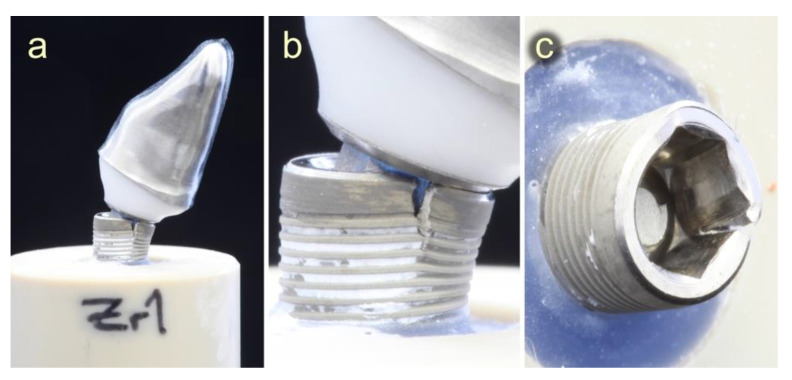
Fracture of the implant after static loading. (**a**) PBJ test specimen after fracture, (**b**) close-up of (**a**): expanded implant shoulder with vertical crack, and (**c**) crack formation along the edge of the internal hex.

**Figure 9:**
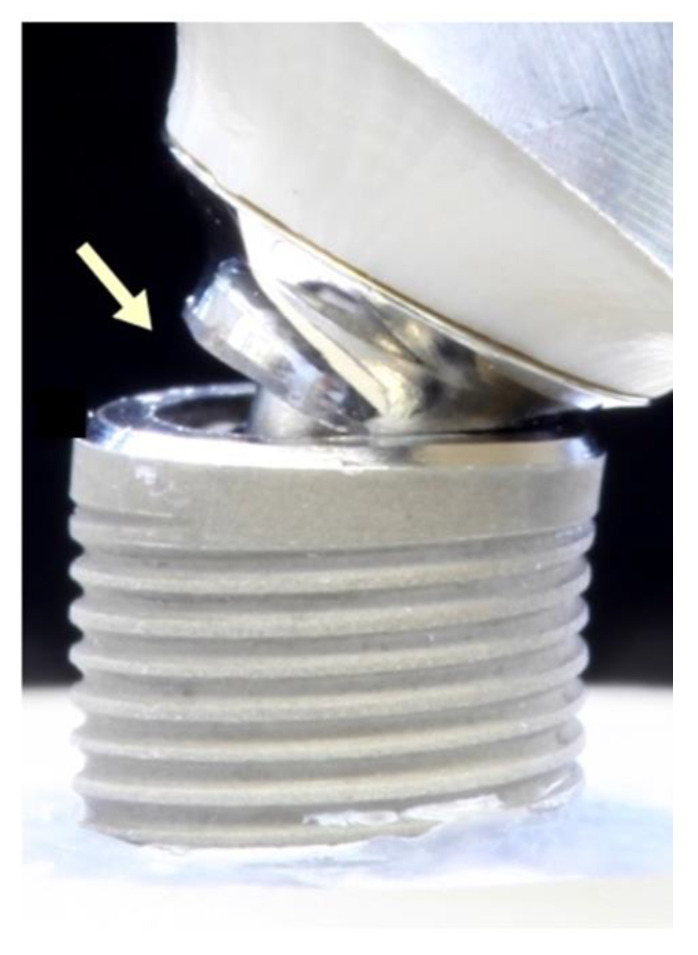
Fracture of the titanium base (arrow) after static loading.

A final static loading test was performed with all experimental specimens that survived dynamical loading (*n* = 23) and their control samples (*n* = 24) which did not undergo dynamical loading. The failure pattern at static loading was similar for all specimens. Failure/fracture of the specimens either occurred in the implant shoulder area (Figure 8a–c) or in the titanium base (Figure 9).

All implants showed an expansion of the implant shoulder in combination with a fracture of the abutment screw. PC specimens showed a uniform failure pattern revealing a bent titanium base and expanded implant shoulder. Solely one sample in the PC group showed a different pattern, with a horizontally fractured titanium base fractured on the tensile side and a slightly deformed implant shoulder. This particular test specimen had the lowest fracture load value (723 N) of all test specimens within the PC group. Similarly, all 32 test specimens with a butt-joint connection geometry (PBJ + ZA) showed a uniform fracture pattern in the area of the implant shoulder, regardless of the abutment material. Similar to the conical test specimens, implants expanded significantly at the implant shoulder, similar to the conical test specimens. In addition, a vertical crack formed along the internal hexagon socket. Table 5 shows the fracture load (N) results. The highest mean fracture load was found in the two artificially unloaded PEEK groups, whereas the lowest mean fracture load was observed in the ZA group (738 N). The artificially loaded groups presented lower mean fracture load values than their unloaded controls, although this finding failed to reach significance (Table 5).

**Figure 10:**
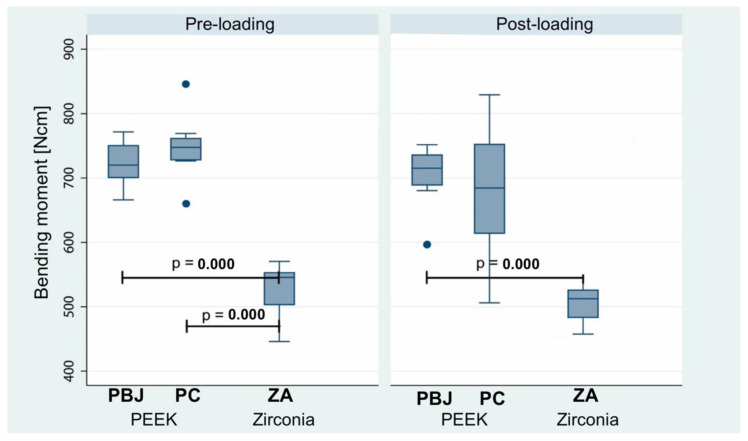
Assessment of bending moments in static loading testing for unloaded and loaded groups. n: number of samples; PBJ: PEEK abutments with an internal butt-joint; PC: PEEK abutments.

Finally, bending moments were determined to account for possible lever-arm differences among groups (Table 6 and Figure 10). Statistical analyses revealed significant differences in bending moments between unloaded groups PC and PBJ versus ZA (*p* = 0.0000), as well as for the loaded groups PBJ versus ZA (*p* = 0.04).

Linear regression model results showed that ZA was generally associated with 195.4 Ncm (95% CI: 166.9–223.9 N cm) lower bending moments than PBJ without considering dynamic loading. Moreover, PEEK abutment geometry (PC vs. PBJ) did not reveal a significant impact on the bending moment.

## 4. Discussion

The present study investigated the physico-mechanical characteristics of PEEK versus zirconia abutments on two-piece titanium-based implants. An additional research focus was set on the impact of PEEK abutment geometries on outcomes assessed with the presented ex vivo chewing simulator model.

Dynamic loading resulted in a 94% survival rate for the PEEK groups and 100% for the zirconia group. When assuming a masticatory rate of 250,000 contacts/year [27], the present ex vivo data translates to satisfying survival rates after a simulation of approximately 20 years of clinical function for both examined materials. Fracture load is an important biomechanical parameter for the long-term performance of implant–abutment constructions. All 48 test specimens in this investigation were loaded to fracture in a static load test in order to be able to classify the fracture load of the PEEK abutments in comparison with zirconia abutments. The inclusion of an unloaded control group further allowed us to evaluate the impact of dynamic loading on fracture resistance. The evaluation of the fracture pattern showed the implant shoulder and the titanium base to be the bottleneck of the systems. When comparing the fracture loads of the study groups, the PEEK groups performed better than the zirconia abutments. The PEEK groups had mean fracture load values of up to 1101 N, whereas the zirconia group reached 772 N. The large difference in elasticity between PEEK and zirconia was probably the main influencing factor for the differences in fracture load. One might assume that the deformation of the PEEK material diverted the acting forces and that the forces acting on the implant shoulder were, thereby, reduced in this process. Stress maxima which arose could, therefore, be absorbed more safely. However, it would require future in-depth research, including biomechanical analysis, such as finite element analysis, to evaluate the material’s behavior and stress distributions during loading.

Preis et al., investigated the long-term resistance of CAD/CAM fabricated implant-supported PEEK frameworks in an artificial chewing simulation and subjected the test specimens to a static loading test. The average fracture load was 921 N, whereas that of the control group (teeth) was 747 N [25]. In addition to PEEK crowns, six other systems made of zirconia and composite were tested. Overall, PEEK exhibited the lowest average fracture loads. The mean fracture load for zirconia was approximately five-fold higher (4817 N) than PEEK. However, compared to the present work, the test specimens were loaded axially and not at an angle of 30°. As a result, there was no leverage subjected to the abutment during loading. The presented values might, therefore, not be comparable with those of the present study. In summary, the authors reported that PEEK abutments veneered with composite performed worse than other examined specimens. In contrast, based on ex vivo testings, the workgroup of Rosentritt concluded that PEEK could be proven up to 0.4 × 106 loading cycles, which translated to 1.5–2 years of clinical application [25]. However, they considered further tests to be necessary and stated that PEEK might be particularly appropriate for application in the anterior zone. With minimum fracture load values of 801 N in the static load test, the fracture load of the PEEK test specimens in our study was above most of the reference values found [23,24,25,46] and above the maximum masticatory forces of 595 N typically found in vivo [47]. 

Regarding the influence of material and geometry on microgap values, it could be shown that ZA was generally associated with lower microgap values compared to the microgaps detected for PEEK abutments. Gehrke et al., measured TAIMs of zirconia hybrid abutments and reported mean values of 45.6 μm [48]. These were higher than the values found for the ZA test group in the present work. The difference in the marginal gap size between PEEK and zirconia could be due to a poorer fit of the PEEK abutments or to the different manufacturing processes of test specimens. Notably, many of the PEEK test specimens showed foreign bodies in TAIMs after the chewing simulation. The marginal gap of the ZA comparison group was closed and filled with an adhesive bond due to the adhesive bonding process. Thus, no particles could be deposited here during the chewing simulation. This suggests that the manufacturing process of PEEK abutments may not allow the marginal gap to be adequately sealed and, thus, entails the risk of a foreign body reservoir during clinical application.

A comparison of PEEK groups showed that slight differences existed for the pre-loading situation, whereas the type of abutment connection did not influence microgap value differences in the post-loading comparison. However, the linear regression model adjusted for timepoint of measurement revealed that conical test specimens generally had 5.4 μm lower IACM values than parallel implant–abutment geometries. A possible explanation for this could be the different dynamics of the assembling process. When screwing the parallel abutments, the contact surfaces are pressed against each other under tension, but both components can easily separate when the retaining screw is loosened. Baj et al. [49] reported marginal gap values of 1 μm to 4 μm for conical connections, whereas Gehrke et al., found values of 0.3 μm to 9.0 μm [49]. Conical test specimens’ IACM values in our experiments ranged from 6.9 μm to 9.2 μm which was in accordance with the data provided by Gehrke et al. [48]. Overall, when ranking our results in the range of the available literature IACM values (0.3–14.3 µm), PBJ IACM values ranked at the upper end (median: 10.1–15.7 μm), followed by PC (median: 6.9–9.2 μm) and ZA (median: 1.7–1.8 μm).

The influence of dynamic loading on IACMs was heterogeneous among experimental groups. For PBJ, IACMs decreased due to the loading procedure from 15.9 ± 4.5 μm to 10.6 ± 5.1, whereas IACMs increased from 6.8 ± 1.5 µm to 9.3 ± 1.7 µm in the PC group. One might assume that this probably occurred due to loading at an angle of 30° and different internal geometries forwarding the applied force differently for both specimens. For parallel abutments, the marginal gap was found in a vertical plane, whereas microgaps appeared in a horizontal plane for conical abutments. This could be considered an explanation for vertical height loss in PBJ and horizontal widening of the implant shoulder in PC. Interestingly, no influence of the chewing simulation on the marginal gaps in the parallel zirconia group compared to PBJ could be found. This might be explained due to higher initial marginal gap widths in PBJ (15.9 μm) compared to the small pre-loading microgap values of the ZA group (2.0 µm). The clinical relevance of the marginal gap might be seen in the potential risk of developing peri-implantitis [50]. It is reasonable to keep the marginal gap width small in order to offer anaerobic bacteria as small a germ reservoir as possible. Overall, further improvements are needed to maintain smaller microgap values for PEEK implant abutments. 

Furthermore, Zirconia was associated with 0.5 µm (95% CI: 0.472–9.571) higher Ra values and 7.6 µm (95% CI: 6.916–8.211 µm) higher Rz values compared to PEEK implant abutments in the present ex vivo model, indicating a favorable PEEK surface property in oral cavities. Additionally, PC showed a significant decrease in surface roughness Ra in 2/4 measurement positions after the chewing simulation. In the analysis of Rz, 4/4 measurement positions showed a significant decline in surface roughness after the chewing simulation. The decrease ranged between 1.6 μm and 8.6 μm. The surface change was possibly caused by constant rinsing with water during thermal cycling, as well as by the cyclical compression of the superstructures during the dynamic chewing load. However, it is notable that the PEEK group PBJ did not show a significant impact of the chewing simulation on Ra and Rz values. Although the abutment geometries could theoretically represent an explanation for the different roughness changes, abutments were generally constructed similarly and came from the same production batch. Therefore, this finding requires further exploration. Notably, there was a significant source of heterogeneity when measuring surface roughness. The main reason lies in different measuring devices with varying levels of precision [51,52]. Overall, although surface roughness measurements may have highly varied in the literature, PEEK seems to perform at least as well as its zirconia comparator when it comes to wear-related surface changes.

The present study is associated with strengths and limitations. To the best of the knowledge of the presenting authors, this is the first ex vivo study on the application of PEEK as a novel definitive implant abutment material. Utilizing the presented ex vivo chewing simulator model allowed us to compare two different PEEK abutment geometries with well-established zirconia abutments. The validity and reproducibility of experiments were great advantages of the applied model, as parts of the experiment were standardized according to ISO standard 14801 (DIN EN ISO 14801). Compliance with standards allowed for the comparison of multiple study outcomes compared to non-standardized ex vivo testing approaches [53]. All applied loading settings in the present study were within the required tolerance range stated in ISO 14801. In order to interpret variable values specified under ISO standard 14801 (modulus of elasticity, bone loss, and loading angle), they should be related to clinical findings. The modulus of elasticity of the PEEK sample holder used in this work was 8 GPa and, therefore, adjusted in the range of cortical bone (7–22 Gpa) [54,55]. Although the simulation of 3 mm bone recession in our approach may have appeared high compared to clinical situations, it took into account more unfavorable clinical conditions [56]. The dynamic load of 49 N selected in this study was based on values from clinical studies reporting mean physiological chewing forces of 24 N to 75 N for the anterior region [57,58]. Furthermore, 49 N is a typical loading protocol allowing future between-study comparability [59,60]. Our approach included static and dynamic loading mimicking the in vivo situation as closely as possible compared to studies performing solely static loading. Availability of multiple testing chambers allowed to test different experimental groups simultaneously with the same experimental condition, thus, reducing potential experimental bias. Another advantage was the inclusion of a control group that underwent solely static loading, allowing the evaluation of the impact of artificial loading/aging on the examined materials. Limitations of the chosen approach included the lack of standardized failure measurements. Failures had to be measured twice a day by one examiner. Thus, exact timepoint determination and documentation of failure occurrence could not be performed. Future sensor- and video-based documentation of failure mechanisms are warranted [61]. Moreover, biomechanical analysis techniques such as 3D finite element analysis could be applied to gain a more in-depth knowledge of stress distributions and failure characteristics within examined materials. Finally, it needs to be mentioned that the PEEK surface is exposed to additional factors (biofilm, discoloration, acids, abrasive particles, etc.) in clinical situations, which could not be taken into account in the present study. Consequently, one cannot conclude the possible degenerative influences of these factors. It would make sense to examine the material stress on the PEEK surface in further experimental models in this context. The wear resistance of PEEK, for example, was reported to be only half that of titanium [62]. A visible material erosion impairing the stability is, therefore, conceivable. Likewise, intrinsic discolorations that interfere with aesthetics cannot be ruled out with examination despite inert surfaces.

## 5. Conclusions

Within the limitations of the present investigation, it could be concluded that PEEK abutments showed superior load-bearing properties compared to zirconia abutments, although, it was associated with greater microgaps at the implant–abutment complex. Parallel and conical PEEK abutment geometries were similarly successful in the mechanical tests. The present study highlighted the feasibility of PEEK implant abutment solutions in modern implant dentistry. Further external validation and expansion of provided evidence are encouraged to finally recommend this material for long-term clinical application.

## Figures and Tables

**Table 1:** Descriptive statistics of implant–abutment connection microgap (IACM) values (in µm) for each experimental group and timepoint (before and after dynamical loading). PBJ: PEEK abutments with an internal butt-joint connection; PC: PEEK abutments with an internal conical implant–abutment connection; ZA: zirconia abutments.

Pre-Loading					
Group	Median	Mean	Standard Deviation	Minimum Value	Maximum Value
PC	6.9	6.8	1.5	3.0	10.9
PBJ	15.7	15.9	4.5	6.9	32.3
ZA	1.8	2.0	1.1	0	5.9
**Post-Loading**					
**Group**	**Median**	**Mean**	**Standard Deviation**	**Minimum Value**	**Maximum Value**
PC	9.2	9.3	1.7	5.3	13.2
PBJ	10.1	10.5	5.1	2.4	21.1
ZA	1.7	2.0	1.4	0	7.6

**Table 2:** Descriptive statistics of titanium base–abutment interface microgap (TAIM) values (in µm) for each experimental group and timepoint (before and after dynamical loading). PBJ: PEEK abutments with an internal butt-joint connection; PC: PEEK abutments with an internal conical implant–abutment connection; ZA: zirconia abutments.

Pre-Loading					
Group	Median	Mean	Standard Deviation	Minimum Value	Maximum Value
PC	43.9	47.2	14.2	22.3	85.9
PBJ	34.5	36.3	11.8	12.9	64.9
ZA	11.1	11.9	8.0	0	38.6
**Post-Loading**					
**Group**	**Median**	**Mean**	**Standard Deviation**	**Minimum Value**	**Maximum Value**
PC	44.9	44.8	10.3	25.7	77.3
PBJ	38.4	38.8	10.2	15.3	67.2
ZA	8.2	11.0	9.7	0.7	46.0

**Table 3:** Descriptive statistics of pre- and post-loading average surface roughness Ra (in µm). PBJ: PEEK abutments with an internal butt-joint; PC: PEEK abutments with an internal conical implant–abutment connection; ZA: zirconia abutments.

Pre-Loading Ra (in µm)					
Group	Median	Mean	Standard Deviation	Minimum Value	Maximum Value
PC	1.2	1.2	0.4	0.5	2.7
PBJ	0.9	1.0	0.2	0.6	1.9
ZA	1.5	1.5	0.4	0.6	3.1
**Post-Loading Ra (in µm)**					
**Group**	**Median**	**Mean**	**Standard Deviation**	**Minimum Value**	**Maximum Value**
PC	1.0	1.0	0.2	0.6	2.3
PBJ	1.0	1.0	0.2	0.7	1.5
ZA	1.5	1.5	0.4	0.7	2.7

**Table 4:** Descriptive statistics of pre- and post-loading average surface roughness Rz (in µm). Rz was the average surface profile maximal height. PBJ: PEEK abutments with an internal butt-joint connection; PC: PEEK abutments with an internal conical implant–abutment connection; ZA: zirconia abutments.

Pre-Loading Rz (in µm)					
Group	Median	Mean	Standard Deviation	Minimum Value	Maximum Value
PC	11.3	12.9	7.2	5.7	68.1
PBJ	8.6	9.0	2.1	6.1	19.0
ZA	16.3	16.6	4.6	6.1	36.9
**Post-Loading Rz (in µm)**					
**Group**	**Median**	**Mean**	**Standard Deviation**	**Minimum Value**	**Maximum Value**
PC	8.9	9.3	2.4	5.5	25.2
PBJ	8.6	8.8	1.3	6.1	13.1
ZA	15.7	16.3	4.6	6.9	33.6

**Table 5:** Fracture load (in N) for experimental and control groups. n: number of samples. PBJ: PEEK abutments with an internal butt-joint connection; PC: PEEK abutments with an internal conical implant–abutment connection; ZA: zirconia abutments.

Unloaded Control Groups						
Group	n	Median	Mean	Standard Deviation	Minimum Value	Maximum Value
PC-NL	8	1100	1101 ^0,a^	86	965	1270
PBJ-NL	8	1055	1056 ^0,a^	48	997	1150
ZA-NL	8	797	772 ^1*,a^	58	652	822
**Loaded Experimental Groups**						
**Group**	**n**	**Median**	**Mean**	**Standard Deviation**	**Minimum Value**	**Maximum Value**
PC-L	8	944	851 ^0,a^	354	49	1200
PBJ-L	8	1045	1018 ^0,a^	68	871	1080
ZA-L	8	748	738 ^1*,a^	42	665	805

In loaded/unloaded groups: same superscript number = groups not significantly different (* *p* = 0.000 unloaded, *p* = 0.048 loaded); a: between loaded and unloaded groups: same superscript letter = groups not significantly different.

**Table 6:** Bending moments (in Ncm) for experimental and control groups. n: number of samples. PBJ: PEEK abutments with an internal butt-joint connection; PC: PEEK abutments with an internal conical implant–abutment connection; ZA: zirconia abutments.

Unloaded Control Groups						
Group	n	Median	Mean	Standard Deviation	Minimum Value	Maximum Value
PC-NL	8	747	747 ^0,a^	52	659	846
PBJ-NL	8	720	722 ^0,a^	35	666	772
ZA-NL	8	546	528 ^1*,a^	41	446	570
**Loaded Experimental Groups**						
**Group**	**n**	**Median**	**Mean**	**Standard Deviation**	**Minimum Value**	**Maximum Value**
PC-L	8	661	596 ^0,1,a^	246	36	829
PBJ-L	8	715	704 ^0,a^	49	597	752
ZA-L	8	512	508 ^1*,a^	33	458	561

In loaded/unloaded groups: same superscript number = groups not significantly different (* *p* = 0.000 unloaded, *p* = 0.04 loaded); a: between loaded and unloaded groups: same superscript letter = groups not significantly different.

## Data Availability

Data supporting the findings of this study are available from the corresponding author upon reasonable request.
